# Information Theory as an Experimental Tool for Integrating Disparate Biophysical Signaling Modules

**DOI:** 10.3390/ijms23179580

**Published:** 2022-08-24

**Authors:** Patrick McMillen, Sara I. Walker, Michael Levin

**Affiliations:** 1 Allen Discovery Center at Tufts University, Medford, MA 02155, USA; 2 Beyond Center for Fundamental Concepts in Science, Arizona State University, Tempe, AZ 85281, USA; 3 Santa Fe Institute, Santa Fe, NM 87501, USA

**Keywords:** information theory, embryogenesis, regeneration, cell biology, morphogenesis, calcium

## Abstract

There is a growing appreciation in the fields of cell biology and developmental biology that cells collectively process information in time and space. While many powerful molecular tools exist to observe biophysical dynamics, biologists must find ways to quantitatively understand these phenomena at the systems level. Here, we present a guide for the application of well-established information theory metrics to biological datasets and explain these metrics using examples from cell, developmental and regenerative biology. We introduce a novel computational tool named after its intended purpose, calcium imaging, (CAIM) for simple, rigorous application of these metrics to time series datasets. Finally, we use CAIM to study calcium and cytoskeletal actin information flow patterns between *Xenopus laevis* embryonic animal cap stem cells. The tools that we present here should enable biologists to apply information theory to develop a systems-level understanding of information processing across a diverse array of experimental systems.

## 1. Introduction

It is an inherent limitation of science that experimentalists are constrained by their tools; however, the phenomena that we study do not share these limitations. When we conflate the constraints of our methodology with the constraints of our systems we lose sight of the importance of what we cannot yet measure. During the 20th century, the establishment of the central dogma and the advances in genetics and molecular biology has enabled us to perturb and quantify biological systems at a microscopic level. During the 21st century, it has become increasingly clear that biology is not so easily reduced to its microscopic component parts, which often, individually, do not retain the fundamental features that animate matter across scales. As we expand our understanding of biology from cells and pathways to tissues and systems, we must move beyond our simple linear ideas of causality such as “signaling pathways” and “genotype determining phenotype” to do justice to the complexity of biological information processing [1,2,3,4].

Three main barriers exist to the rational discovery of interventions based on simple systems-level causal models in cell and developmental biology. First, there is the problem of time. Many manipulations, including drug treatments and genetic knockouts, constitutively activate or inhibit specific pathways but in so doing remove any information encoded in the dynamics of the signal. There is a growing body of evidence that such dynamics are crucial to proper cell signaling with specific outcomes encoded by different signal dynamics [5,6,7,8,9]. Importantly, temporal interactions are not merely inconvenient artifacts, but core to the very idea of causality [10,11]. Emerging tools such as optogenetics [7,12] have allowed us to manipulate systems with high temporal resolution, but new analytical approaches are necessary to implement these tools at the systems level, in both space and time, in non-neural systems.

**Figure 1:**
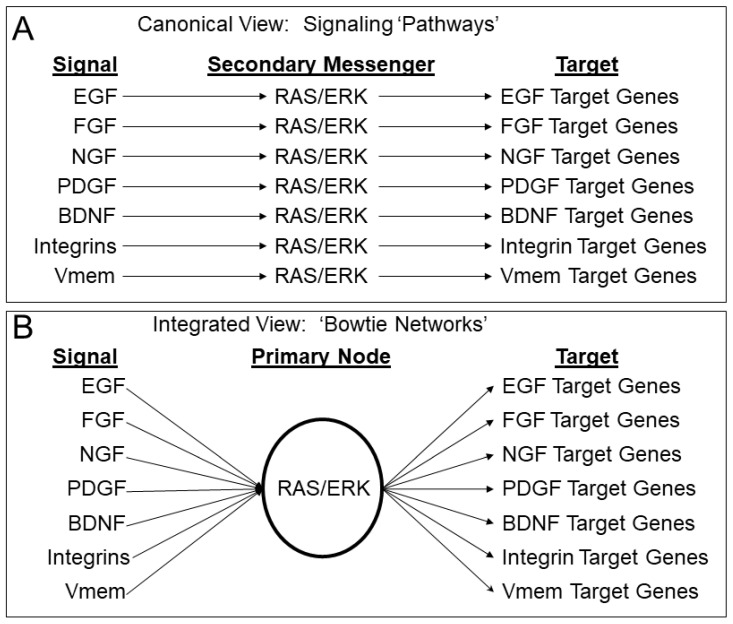
Secondary messengers as primary nodes: (**A**) Cell signaling is often conceptualized as pathways between signals and target genes with secondary messengers as intermediates; (**B**) because the same secondary messengers are used in many different pathways, it may be more appropriate to think of them as primary nodes integrating diverse signaling regimes.

Second, there is the problem of integration. Due to technical limitations, it is standard to manipulate a single pathway at a time and test for effects on a targeted set of outcomes. However, biological systems are built to be robust to just this type of insult, manifesting as spontaneous mutations, and thus, we are often limited in our ability to understand and control systems governed by redundancy and integration. Further, the specificity of independent “pathways” is confounded by the tendency of multiple different pathways converging into a small set of “secondary messengers” (Figure 1) [13,14,15,16,17,18,19]. Understanding how diverse inputs are integrated would dramatically improve our ability to predict and control biological systems, but would require supplementing beyond classical reductionist methodology with new integrative approaches.

Third, there is the problem of computation. It is clear that studying an individual neuron is entirely insufficient to understand the functioning of the nervous system [20]. Neuroscientists overcame this problem by applying tools such as positron emission technology (PET), fMRI, and calcium imaging to measure systems-level behavior of neural tissues. In non-neural systems, it is also becoming increasingly clear that many aspects of tissue-level function are irreducibly complex and require new tools that go beyond simple linear pathways commonly used in molecular genetics models [21,22,23,24]. The above techniques are increasingly being adopted by non-neural biologists, but without analytical tools to deal with the data that they produce that cannot be used to their fullest potential.

Here, we propose applying information theory as a useful approach to these three problems. Information theory provides the methodology for quantifying correlative and causative interactions between different variables. Though it forms the basis of computation, information theory is not to be confused with mathematical modeling. Indeed, information theory is unique in being a model-agnostic method with respect to the implementation details of the system being analyzed. It can readily be applied to extremely different systems, including gene–gene interactions, genotype–phenotype interactions to cell–cell interactions and beyond, requiring very few prior assumptions of how the systems work (i.e., being compatible with a wide range of mechanisms). By allowing researchers to map information flow between cells and pathways, information theory shows promise to facilitate systems-level understandinga of biological phenomena for which molecular reductionism has failed, and to leverage the emergent power of tissues for synthetic biology applications [22]. Indeed, the transformative nature of information theory should not be underestimated; Shannon’s 1948 paper [25] directly ushered in the information age and the global integration that defined it, and his formalism is the basis for the analysis that we present here.

**Figure 2:**
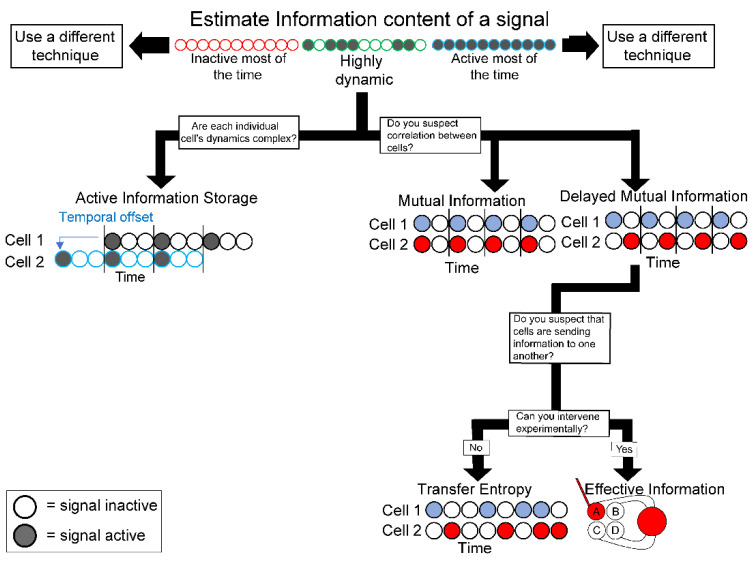
Information metric flowchart. Suggested workflow for deciding which information theory metrics to apply based on qualitative observations of a system.

Here, we describe useful information theory metrics and provide analogies and examples from known biological systems. Our goal is to describe a variety of information theory techniques and to illustrate how they could be applied by experimental biologists (Figure 2). We explore concepts with analogy and re-evaluation of experimental systems, but do not delve too deeply into the underlying mathematics of these concepts, since each is a well-accepted technique supported by rigorous mathematical principles that are described in detail in the cited references.

Then, we introduce a novel tool, CAIM, designed to allow biologists to easily and rigorously apply several of the metrics we discuss to their own data. We demonstrate how CAIM can be applied to describe information flow dynamics at the systems level of calcium, a key signaling node, and actin, a key component of the cytoskeleton that directly effects many biological processes including morphogenesis, the early embryonic *Xenopus laevis* stem cells. Finally, we integrate these data to propose a model of the systems-level functions of these signaling modalities in this primitive tissue.

### 1.1. Information Metrics

**Figure 3:**
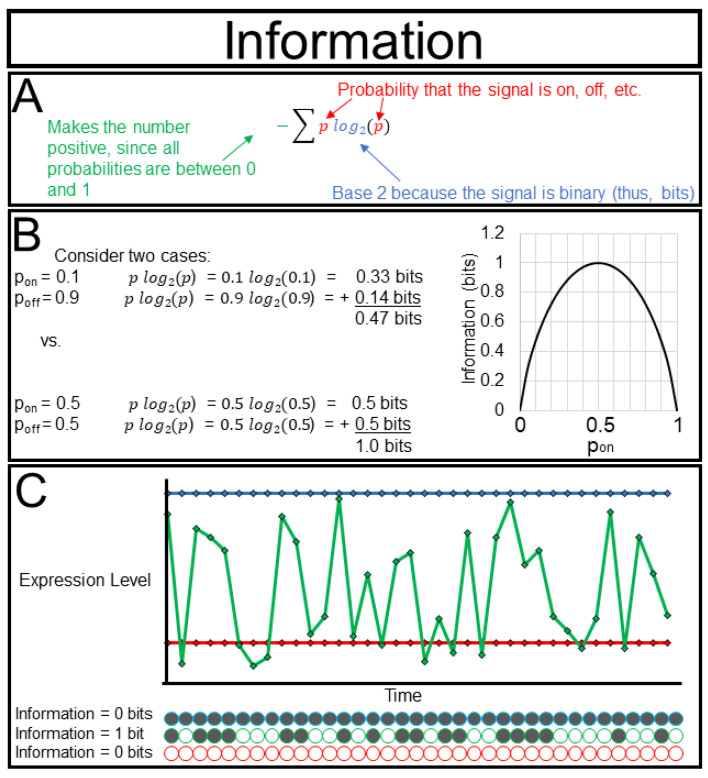
Information: Information is a formally defined value (**A**) that increases as variables spend more time in multiple states (**B**). Signals that frequently shift between ”active” and ”inactive” states (green) contain more Information than signals that are either always on (blue) or always off (red) (**C**).

The real power of information theory comes from its rigorous mathematical foundation. Science is fraught with beautiful ideas such as ”memory”, ”consciousness”, and even ”life” that seem intuitively clear but are too nebulous to withstand rigorous scientific scrutiny. The concept of information is among these in some senses, but in others its meaning is not debatable. This is true where concepts of information have been mathematically formalized. In 1948, Claude Shannon famously defined information using the formula I = − Σplog(2)p, with I denoting the information content of a signal and p being the probability that the signal is active, and which we describe in greater detail in Figure 3A. This formula may seem familiar, and indeed it is very similar to the formulation of thermodynamic entropy. The two concepts are closely related. Consider two libraries, one library that is well-organized (low thermodynamic entropy) and the other library that is very cluttered (high thermodynamic entropy). Describing the location of every book in a well-organized library requires very little information (“they are alphabetical by title starting here”), while describing the location of every book in a disorganized library requires a lot of information (“Melville is there, Shakespeare over here, Seuss down in the basement” etc.) Just as thermodynamics allows us to quantify and apply a seemingly nebulous concept such as ”order”, information theory allows us to quantify reduction in our uncertainty about the state of a system, with respect to a specified syntax or language.

Quantifying information can aid in one of the greatest challenges for the experimentalist, i.e., deciding which tool to use to study a system and what intervention to perform. Imagine that we have three fluorescent reporters for the same metabolite with different sensitivities (Figure 3C). The first reporter is active 10% of the time (I = 0.47 bits), the second reporter is active 90% of the time (I = 0.47 bits), the third reporter is active 50% of the time (I = 1.00 bit). Even though we obtain the most signal from the second reporter we obtain the most information from the third. Most of the metrics that we describe below are bounded by the amount of information of a variable, and thus, it is wise to tailor our approaches to extract the maximum amount of information from our system.

#### 1.1.1. Mutual Information (MI)

**Figure 4:**
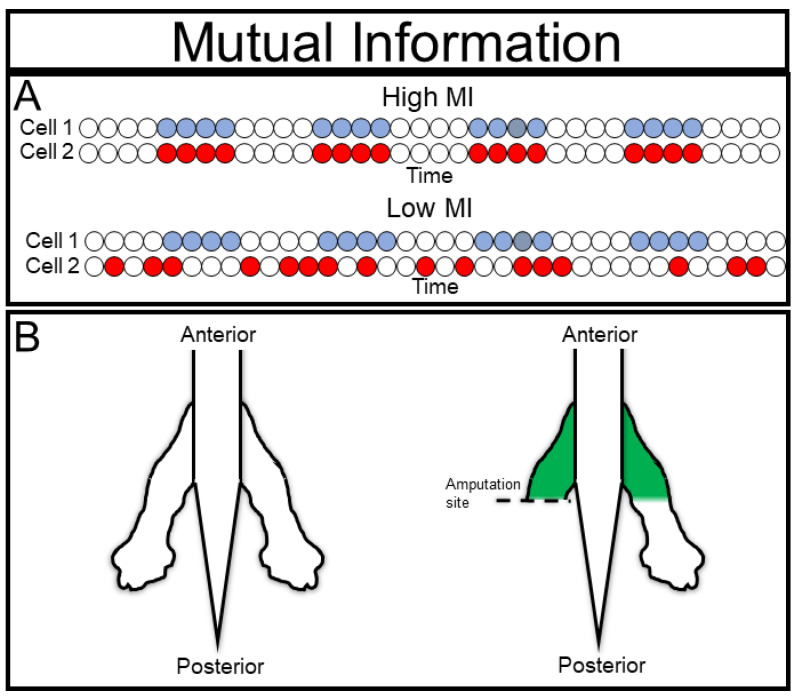
Mutual Information: (**A**) Mutual information measures the information that knowing the state of one variable provides about a second variable; (**B**) mutual information can be used to detect novel communication channels, as in the case of contralateral bioelectric injury signals. Redrawn with permission after [26]; 2018, The Company of Biologists Ltd.

Mutual information (MI) is a measure of how much our uncertainty of one variable is decreased by knowing the state of another variable [25] (Figure 4A). Imagine that Alice and Bob always arrive to work at 8:00 a.m. The arrival times of these people will have very high mutual information, i.e., we can usually tell if Bob is around by knowing whether or not Alice is around. We cannot, however, predict anything about the relationship between Alice and Bob. It may be that they live together and carpool; it may be that they live separately but arrive on the same bus; or it may be that both of their shifts start at 8:00 a.m. MI does not provide any insight into the causal reason(s) that the two arrive at the same time.

From a practical standpoint, establishing mutual information between two factors can be very important even without knowing the mechanistic interaction between the two factors, especially if one of the factors is difficult to measure. This is the core of what physicians do in diagnostic assays, i.e., measure phenomena with high mutual information to disease states. It is not necessary to understand why heart attacks cause intense pain in the left arm, it does not really matter if we can reliably use the high MI between the two to quickly diagnose heart attacks. A biological example of this utility from our own work can be seen in symmetrical effects of amputation [26]. We observed that upon amputating a developing frog limb, the contralateral (undamaged) limb would stain strongly with the potentiometric dye DiBAC_4_(3) at approximately the proximal-distal location as the site of amputation, consistent with these two distant sites having surprisingly high mutual information post-amputation (Figure 4B). The cells of one limb had information about the state of cells in the opposite limb; this finding revealed an unexpected phenomenon even before investigations of the mechanism of the correlation, for example, suggesting the possibility of surrogate-site diagnostics.

As a warning to the experimentalist, while MI is useful as an initial guide to forming hypotheses, as it can also reveal relationships that are “artifacts” in the sense that they do not represent interesting causal connections. For example, if the light source used in a microscopy experiment fluctuates over time, then two nearby cells will demonstrate high MI, but this MI will not reflect biologically meaningful interactions. Despite these caveats, MI is a powerful ”first step” for information theory analysis because its model agnostic nature facilitates discovery of unexpected phenomena.

#### 1.1.2. Delayed Mutual Information

**Figure 5:**
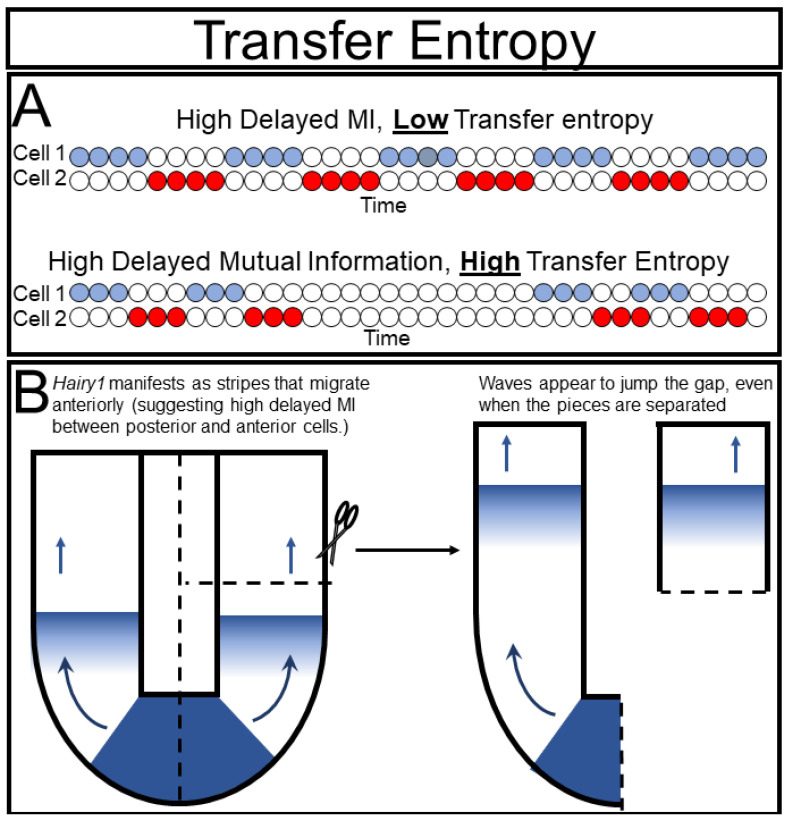
Delayed mutual information and transfer entropy: (**A**) Delayed mutual information measures the predictive power of one variable to another variable’s future, allowing for detection of causal interactions, whereas, transfer entropy is a more powerful approach that considers predictive power of the receiving variable’s own history, and thus, avoids inferring causation from time delayed correlative interactions; (**B**) the vertebrate segmentation clock is a biological example of the incorrect interpretations that can arise from failing to account for a cell’s history. Waves of gene expression appear to migrate posterior to anterior in the growing tail, but will form even when the apparent ”sending” cells are ablated, indicating that they result from intrinsic oscillations within the cells.

An obvious limitation to the applicability of mutual information is its inability to quantify events that are highly correlated but separated in time. This prevents MI from detecting causal interactions in which, by definition, one event must precede another. A simple solution to this problem is to temporally offset the expected ”sending” variable from the ”receiving” variable. If it takes a signal four second to propagate from Cell 1 to Cell 2, then measuring the state of Cell 1 should accurately predict the state of Cell 2 four seconds later (Figure 5A). Returning to the example of the beating heart, imagine two people with similar resting heart rates. Assuming their heart rates are not in phase, there will be a high degree of delayed MI between the two hearts; we can accurately predict when Person A’s heart is going to beat if we know when Person B’s heart beats.

An example of the potential pitfalls of delayed mutual information can be found in the elegant experiments of Isabel Palmeirim and colleagues in their discovery of the segmentation clock. They noticed waves of hairy gene expression that seemed to emerge from the tip of the tail and move anteriorly until they eventually formed a new body segment [27]. In information theory terms, they essentially detected high delayed mutual information between cells along the anterior-posterior axis, which suggested that the posterior cell was directly causing the state of its anterior neighbor. They cleverly tested this hypothesis by ablating the “sender” cells in the tailbud (Figure 5B). Surprisingly, the hairy wave still manifested, demonstrating that the causal prediction from their delayed mutual information analysis was incorrect and inferring that the two cells were oscillating indepyendently. Interestingly, future work [28,29,30] showed that these cells did require some input from their neighbors to oscillate correctly. From this example it is clear that more refined tools are necessary to satisfyingly measure causality, especially in biological systems that cannot be intervened upon as readily as the segmenting tailbud.

#### 1.1.3. Active Information Storage (AIS)

The segmentation clock example above exemplifies the importance of knowing the mutual information between a cell’s current and past states. If a cell’s state is heavily informed by its past this suggests that it is independent and less governed by collective dynamics. We can measure this by measuring the DMI between a cell’s dynamics and a time-delayed version of these same dynamics to generate our next metric, i.e., active information storage.

**Figure 6:**
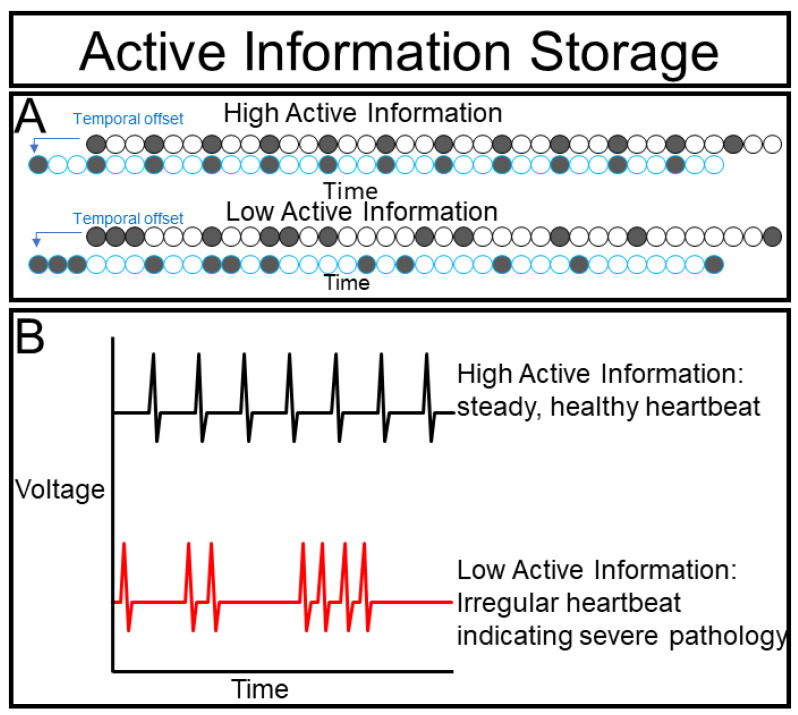
Active information storage: (**A**) Active information storage measures how well a variable’s past predicts its current state; (**B**) a healthy heart should have relatively high active information storage, while an unhealthy heart in which periodicity is pathologically perturbed show have lower AIS.

Consider a beating heart (Figure 6A), we can very easily predict when contractions are going to happen by observing the previous beating rate. Indeed, periodic signals manifest throughout biology, from the vertebrate segmentation clock to circadian rhythms [31]. Active information storage (AIS) is a formulated form of MI between a time series and a past version of itself [32,33,34,35].

Moreover, quantifying changes of AIS can provide important insights into the behavior of a biological system. If the AIS of a beating heart drops slightly, this may indicate that its host organism is exercising or experiencing stress that gradually changes the beat frequency (Figure 4B). Likewise, if the AIS drops in a non-cardiac cell, this loss of autonomous predictive power suggests that the cell is being affected by an external force. We cannot infer what is causing this change, but by identifying and quantifying this interaction we can design experiments to determine its molecular mechanism. If the AIS of a heart drops dramatically this may suggest a serious pathology such as arrythmia or even fibrillation. In turn, dramatic loss of AIS in a non-cardiac cell may suggest an important transition. During differentiation, a cell may become fundamentally different from its previous state, thus, reducing the predictive power of its past for its future. AIS can also drop dramatically if a cell is integrated into a super-cellular collective, as the collective may have substantial input on the state of the cell and only observing the past states of the cell may lose predictive value.

High AIS values, therefore, may suggest that cells are behaving more independently and less collectively. If this is true, then, experimentally isolating the cells should have a minimal effect on the dynamics. Low AIS values, in contrast, suggest that the dynamics are being largely governed by other forces that can be investigated by our next metric, i.e., transfer entropy.

#### 1.1.4. Transfer Entropy (TE)

Inferring causality is one of the main goals of experimentation, but can also be one of the most challenging. Our standard approach is to perturb the system and see how it changes, and then, to attribute the changes to the perturbation. However, these perturbations are often of non-physiological magnitude and, despite our best efforts at control, cn be confounded with technical artifacts. Alternatively, we observe a system over time without perturbation and observe the timing of events, effectively estimating the delayed mutual information between them, but this approach is prone to conflation of correlation with causation. In information theory, the best current tool for inferring statistical causation is called transfer entropy (TE) [36,37]. Recall that the terms entropy and information are often used interchangeably, and thus, TE can be thought of as transfer of information. TE is the amount of information about one variable gained from knowing the state of a second variable beyond what is learned from the history of the first variable (Figure 5A).

Let us say that Alice always arrives to work at 7:50 a.m. and Bob always arrives at 8:00 a.m. It may well be that Alice and Bob drive in together, Alice lets Bob off at the entrance (on account of his bad knee) and goes to park. It may be that Bob hates Alice, and always makes sure to arrive later than her to avoid running into her. In both of these cases it would be fair to say that Alice’s arrival time causes Bob’s arrival time. However, it could also be that Bob just shows up at 8:00 a.m. because that is when his shift starts. Because we can predict when Bob is going to arrive without knowing anything about Alice we cannot really conclude that her arrival time is causing his arrival time, despite reliably preceding it and allowing us to accurately predict.

While transfer entropy is a powerful tool, its broad definition of ”causality” may ring hollow to an experimentalist. We spend our lives intervening upon systems and measuring outcomes to infer causal relationships. TE excels at analyzing systems that cannot be readily manipulated, and can help us decide which systems warrant development of experimental interventions or translation into tractable model systems. Thus, TE allows identifying targets that may be confirmed as causal, once experimental intervention is applied. Once we can manipulate our system, we can move to our next metric, i.e., effective information.

#### 1.1.5. Effective Information (EI)

**Figure 7:**
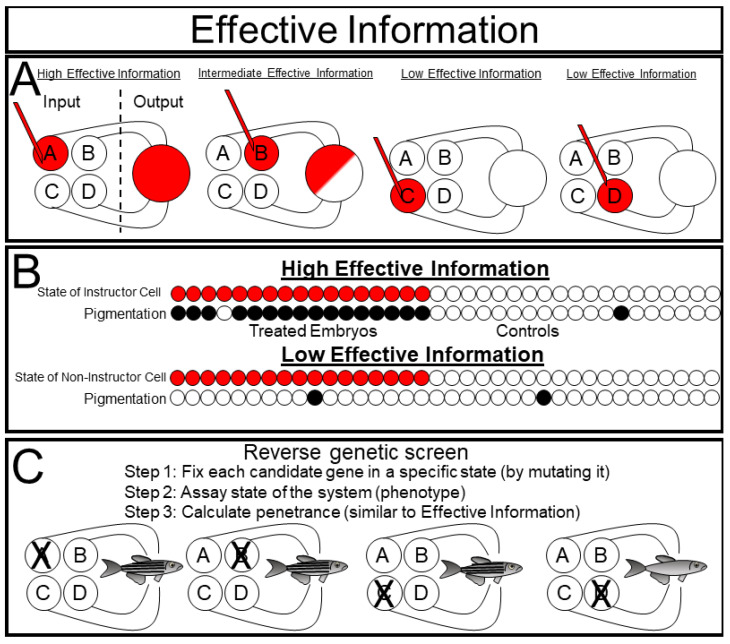
Effective information: (**A**) Effective information measures the predictive power of interventions over a system; (**B**) when ‘instructor cells’ are set to a particular state, the state of the system can be well predicted, and thus, these cells have high effective information with the system, setting the state of non-instructor cells, however, does not well predict the state of the system, indicating low effective information between the cell and the system; (**C**) an approach similar to effective information is used in reverse genetic screens in which each candidate gene (variable) is set to a specific state via mutation and the predictive power of this mutation over a trait is calculated.

Effective Information (EI) is a formal way of measuring causal interactions between an experimental manipulation and a system [17]. To measure EI, one sets the state of each variable and measures the consequences on the future states of the system (Figure 7A). This technique is conceptually very similar to many experimentally implemented biological approaches. In a reverse genetic screen, one individually mutates a set of genes (thus setting their state to 0 for null mutations, <1 for hypomorphic mutations, and >1 for hypermorphic mutations) and observes the phenotypic consequences on the phenotype of the organism (Figure 7C). Importantly, there is rarely a linear one to one correlation between a single gene and the phenotypic outcome of a complex biological system. Biologists refer to this phenomenon as ”incomplete penetrance”.

The discovery of ”instructor cells” in the skin of *Xenopus* embryos provides a nice example of a system with effective information (Figure 7B). Depolarizing a specific subset of epidermal cells causes the entire embryo to become hyperpigmented [38]. Interestingly, this hyperpigmentation exhibits a binary ”all-or-nothing” pattern within each animal, indicating that depolarizing the instructor cells affects the system as a whole [39,40]. In information theoretic terms, they measured the EI between the ”instructor cells” and pigmentation of the embryo system by injecting noise into the instructor cells by either depolarizing them or leaving them as unmanipulated controls, and then estimating the mutual information between the state of the instructor cell and the state of the pigmentation. If they had chosen to depolarize a set of ”non-instructor cells” instead, they would have found very little mutual information between the state of the manipulated cells and pigmentation since the state of the non-instructor cells does not predict the pigmentation state.

Importantly, EI, as with all the metrics presented here, is a continuous, not binary, metric, and thus gives us a framework for quantifying the degree of causal relationship between a manipulation and a phenotypic output. This quantification has the potential to address a substantial problem that has arisen from molecular reductionism, i.e., different researchers apply different methods to similar questions, and subsequently report any statistically significant findings regardless of effect size. Then, these findings are compiled in pathway diagrams in which each experiment is recorded as an arrow, and all arrows are weighted equally regardless of whether the manipulation caused a 10% change or a 90% change and regardless of the technical differences among the techniques used. Because of its model-free nature, EI has the potential to unify these diverse experimental findings in terms of their measurement in bits, which, then, can be compared and compiled.

Consider two researchers studying the same protein. One researcher finds that an intervention increases phosphorylation of protein X 80% of the time via Western blot. The other researcher finds that the same intervention induces ectopic tails 5% of the time in mice. It is tempting to combine these data to say that ”this intervention increases protein X phosphorylation and causes phenotype Y”, but doing so strips the data of important causal information. EI can be applied just as readily to phosphate groups and tail numbers, and thus allows us to integrate these orthologous techniques.

### 1.2. Information Theory Analysis of Calcium and Actin Dynamics in Xenopus laevis Stem Cells

#### 1.2.1. Secondary Messengers and Primary Nodes

Neuroscientists during the 1990s faced the same fundamental problem that non-neural scientists are facing today: How can we measure information dynamics between cells when the mechanisms that transmit this information are fast and subtle? Rafael Yuste and colleagues solved this problem by effectively using calcium signaling as a proxy for action potentials [41,42,43]. Calcium also functions as an important secondary messenger in many diverse non-neural systems [13,44,45,46,47]. The approach of applying information theory (IT) metrics to non-neural calcium signaling is further supported by the observation of striking long-range coordinated calcium patterns that move across many different cells [48]. Information theory measures states of variables, and as discussed in the preceding section, it is convenient for our purposes to coarse grain the complexity of a cell to a single binary on or off calcium state signal (Figure 3C). Once interactions have been identified and measured, we can employ our prodigious arsenal of molecular tools to determine the molecular mechanisms and functional consequences of these patterns.

It is unreasonable to assume that calcium levels alone are sufficient to define the state of a cell. Fortunately, the factors that make calcium an appealing cell state reporter can also be found in other secondary messengers. The RAS/ERK signaling pathway, as one example, also integrates input from many different signaling pathways and displays large scale emergent dynamical patterns [49,50]. Indeed, it may be time to consider promoting these ”secondary messengers” to ”primary nodes” that do not merely transduce signals but integrate information from many different input pathways to determine the behavior of a cell.

There is a growing body of evidence that pulsatile dynamics in the actin cytoskeleton play important roles during morphogenesis [51]. These cytoskeletal dynamics have been described in a variety of tissues, including the *Xenopus laevis* primitive animal cap embryonic stem cell tissue [52]. Further, pulsatile actin dynamics have been linked with calcium dynamics in several developmental systems including mast cells [53] and the developing *Xenopus* neural plate [54]. In order to demonstrate the profound flexibility of information theory we apply these metrics to both calcium signaling and actin pulsation. These signaling modalities are very different at the mechanistic level. Calcium is an ion that mediates communication through several signaling pathways, while actin is a structural cytoskeletal protein that contributes to a cell’s shape and facilitates its mechanical interaction with its environment [44,51]. We have chosen to analyze these very different signaling modalities to demonstrate how the model-free nature of information theory allows us to study interactions among diverse biological phenomena. We do so in explant *Xenopus laevis* animal cap embryonic stem cells. These cells demonstrate both endogenous actin and calcium dynamics, but it remains unclear how they interact at the systems level and what function, if any, they play during early development. By applying information theory to the dynamics of these signaling modalities between and within minimally manipulated tissue explants, we aim to explore the degree to which these cells behave collectively and what roles these modalities play in this collectivization.

#### 1.2.2. CAIM (Calcium Imaging)

**Figure 8:**
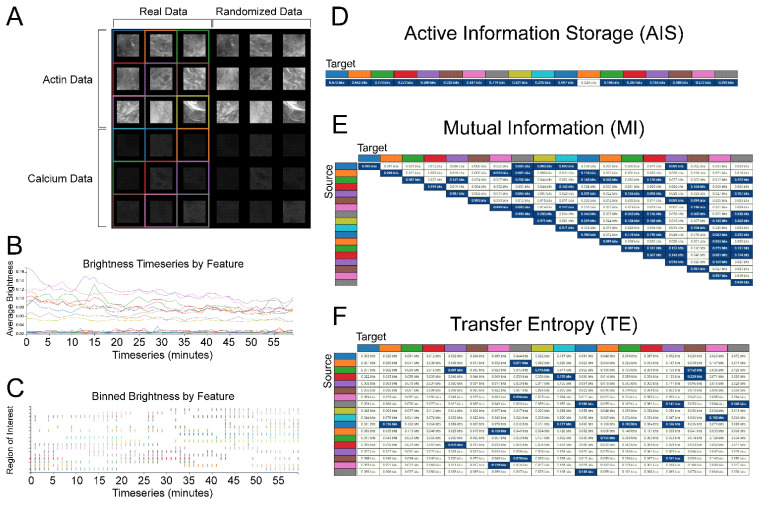
CAIM: A tool for applying information theory metrics to time series data: (**A**) CAIM provides users a GUI to select regions of interest (demarcated by consistent colors throughout the analysis) from which it will extract timeseries data (**B**) that can then be binarized (**C**); (**D**–**F**) then AIS, ME, and TE, respectively, can be calculated between each ROI and displayed in tables with statistically significant values indicated in blue.

Here, we introduce and apply a novel software package called CAIM (calcium imaging) designed to calculate AIS, MI, and TE using the Inform library [55,56]. CAIM was designed as a collaborative effort between biologists and physicists to allow researchers to easily and rigorously apply information theory to real biological datasets. CAIM takes time series datasets (Figure 8A) and plots intensity vs. time for ROIs that can be drawn within the CAIM program (Figure 8B). We generated mosaic time series in FIJI with nine ROIs in four quadrants: real actin data in the top left, real calcium data in the bottom left, randomized actin data in the top right, and randomized calcium data in the bottom right. This setup allows for an easy analysis between different conditions.

CAIM enables binarization of time series datasets via a variety of thresholding techniques and displays the binarized time series (Figure 8C). This function enables researchers to easily assess how accurately the applied binarization represents their data. For our analysis, we have elected to binarize our time series using the mean threshold as a cutoff between signal and noise. Such binarization is not strictly necessary for information theory time series analysis, however, another tool called the Java Information Dynamics Toolkit (JIDT) can be used for continuous datasets [34] but is beyond the scope of this study.

Once the signal is binarized, CAIM can be used to calculate AIS, MI, and TE between ROIs. The results are displayed in tables with individual ROIs being identified by the color used to demarcate them on the time series window. Because AIS does not involve comparisons between conditions, a single value is calculated per ROI. Pairwise comparisons are made between each ROI for MI and TE. Because MI is not directional, only a single value per pair is displayed. Measurement of an ROI’s MI with itself provides the information content of the timeseries as defined by the binarization conditions. As TE is directional, CAIM produces a separate value for each direction of each pairwise comparison. Source ROIs determine the row of the table and target ROI determines the column. Each data point is statistically compared against a randomized group of 1000 random time series and values with *p* < 0.05 colored blue. This provides an easy way to visually detect trends.

#### 1.2.3. Technical Challenges

Due in part to its model-free nature, an information theoretic analysis does not readily distinguish between biologically interesting signal fluctuations and technical artifacts. Strong photobleaching, for example, causes all regions to decrease signal in parallel, and such time series have high mutual information that does not reflect biological behavior. Much of the jGCaMP8S data that we present here rely on comparatively weak subthreshold fluctuations that may be substantially affected by imaging noise which may be registered as signal due to our mean threshold binarization approach. Further, these images were collected using single-plane confocal microscopy, which provides relatively fast clear images, but only visualizes a single surface of the cell, and thus, may miss important factors outside the imaging plane. These potential confounding factors are limitations to be kept in mind and addressed in future work.

## 2. Results

### 2.1. Active Information Storage (AIS): Both Actin and Calcium Dynamics Are Affected by Their Previous States

**Figure 9:**
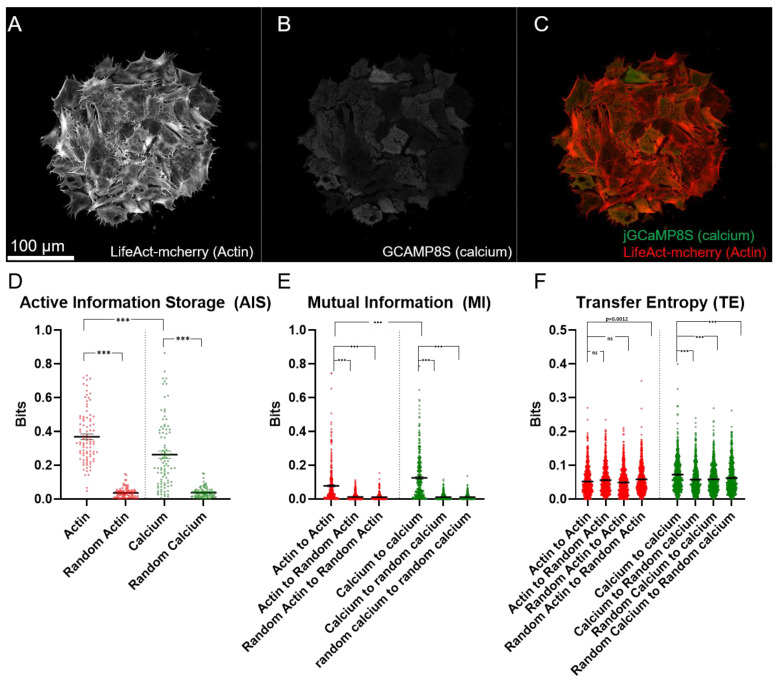
Information metrics within each signal. Representative frame of: (**A**) LifeAct-mcherry; (**B**) jGCaMP8S; (**C**) an overlay of two channels from one of the explants imaged; (**D**) AIS is significantly higher in real vs. randomized datasets for both the actin and calcium signals, and AIS for the actin signal is significantly higher than for the calcium signal; (**E**) mutual information between ROIs within the same time series is higher than for the randomized datasets, and MI is higher between ROIs for the calcium signal than for the actin signal. The calcium signal, but not the actin signal, demonstrates significantly higher TE than any of the randomized datasets (**F**). The real actin signal data demonstrates significantly less TE than the randomized actin does with itself. The real calcium signal also demonstrates significantly greater TE than the actin signal. *** = *p* > 0.0001, Mann–Whitney test.

We measured the active information storage (AIS) of actin and calcium dynamics in each of our ROIs as compared with randomized control time series (Figure 9A–D, Supplemental Movies S2–S4). We imaged nine ROIs on each of 10 explants which were collected from three separate rounds of injection. For both signal types, AIS is significantly higher than the randomized control, suggesting that both actin and calcium dynamics are informed by their previous behavior. This conclusion is consistent with our qualitative observations. Actin pulses seem to be somewhat consistent in duration with a refractory period between pulses. The calcium dynamics, in contrast, seem to change gradually over time, but in both cases the future behavior of the signal is well predicted by its past.

Interestingly, actin dynamics have significantly higher AIS than calcium signaling. This finding suggests that actin dynamics are better correlated with their own history than are calcium dynamics.

### 2.2. Actin and Calcium Dynamics Both Correlate between Nearby Cells

To quantify correlative relationships within actin and calcium dynamics in nearby cells, we measured pairwise MI between all combinations of nearby cells in each of our explants for both channels imaged (Figure 8A and Figure 9E). Then, we compared these values with random datasets generated by measuring MI between these ROIs and randomized ROIs generated from the same explant. We further measured MI between the randomized ROIs. The calcium and actin dynamics both showed significantly increased MI as compared with the randomized datasets generated from their respective channels. Moreover, calcium dynamics showed significantly increased MI as compared with actin dynamics. These data demonstrate that information is shared in both the actin and calcium dynamics of nearby cells, and suggest that more information is shared between cells via the channel of calcium signaling than via the actin dynamic signaling.

### 2.3. Calcium Dynamics, but Not Actin Dynamics, Demonstrate Information Transfer between Nearby Cells

To quantify causal relationships within actin and calcium dynamics, we measured pairwise TE between all combinations of nearby cells in each of our explants for both channels imaged (Figure 9F). Because TE, unlike MI, is directional, we measured TE in both directions for each cell pair. We also generated three random datasets: one dataset in which the sending cell time series was randomized, one dataset in which the receiving cell time series was randomized, and one dataset in which both were randomized.

We did not detect any significant difference in TE between the real actin dynamics dataset and either the ”randomized sender” or the ”randomized receiver” group, and the real data contained significantly less TE (*p* = 0.0012, Mann–Whitney test) than the ”random sender and random receiver” group. Thus, we conclude that there is no detectable transfer of information from one cell to another in the actin signal.

In contrast, we do detect significantly higher TE in the real calcium dynamics dataset than in either of the three randomized controls. This finding suggests that information may be passed from one cell to another cell via the calcium channel but not the actin channel.

### 2.4. Actin and Calcium Dynamics Share Information

Having measured MI and TE between cells for both channels, next, we sought to measure the information dynamics between channels. We began using the same approach that we used for the individual channel analysis, i.e., we measured pairwise MI between the two channels for each pair of ROIs (10 A). We found significantly higher MI between real actin and calcium dynamics than randomized versions of these datasets, although the MI between channels was significantly lower than the MI within the same channel between ROIs. Taken together, these data suggest that actin and calcium dynamics are not independent in animal cap explants.

### 2.5. Actin Dynamics Have Significant TE to Calcium Dynamics, but Calcium Dynamics Do Not Have Significant TE to Calcium Dynamics

**Figure 10:**
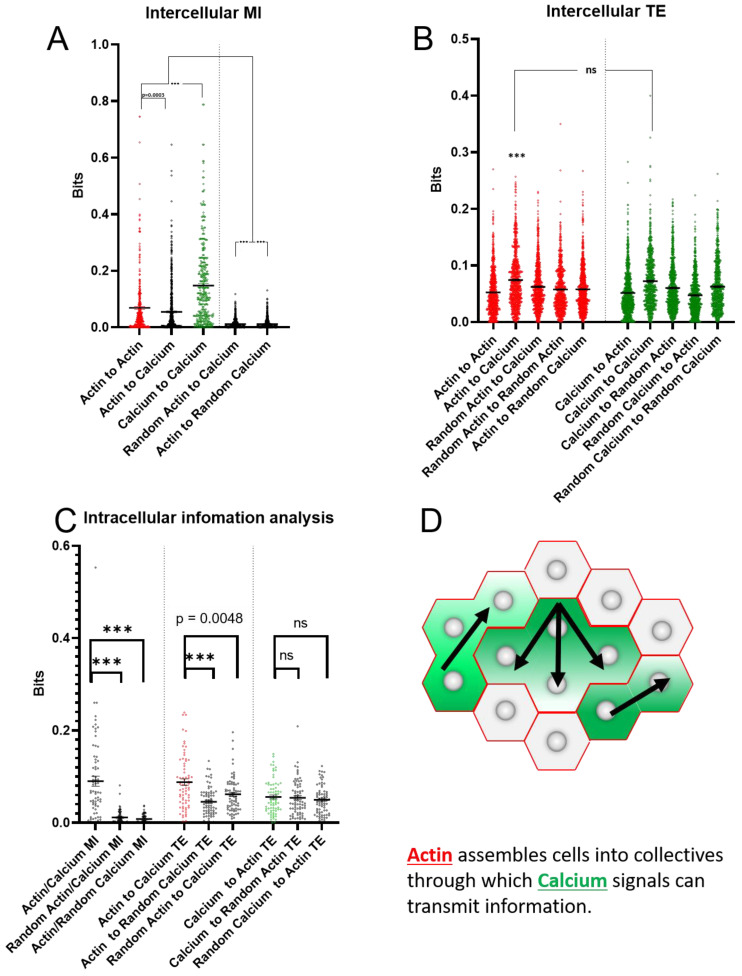
Information metrics between the calcium and actin signals. MI between the actin signal of nearby ROIs, between the calcium signal of nearby ROI, and between actin and calcium signal of nearby ROIs is significantly higher than MI between the randomized actin signal and calcium or the randomized calcium signal to actin. (**A**) The highest MI is observed between nearby ROIs within the calcium signal, with MI between nearby ROIs for the actin signal being significantly higher than MI between the two signals; (**B**) within nearby ROIs, both the actin and calcium signals have significant TE to the calcium signal as compared with randomized controls, with the actin and calcium TE to calcium being statistically insignificant, neither has significant TE to the actin signal; (**C**) within a single ROI, the actin and calcium signals have significant MI, but while the calcium has significant TE to calcium the reverse is not true. From these data we propose a model in which actin establishes boundaries through which calcium signals flow (**D**). *** = *p* > 0.0001, Mann–Whitney test.

We followed our inter-channel MI analysis by measuring TE between pairs of ROIs. We found that actin dynamics had significant TE to calcium dynamics, but that this TE was not bidirectional (Figure 10B). This was somewhat surprising, as we had previously found that actin did not have significant TE between cells while calcium did. This data suggest a complex, directional relationship between actin and calcium in animal cap explants.

### 2.6. Intracellular Mutual Information (MI): Actin and Calcium Dynamics Share Information

To more highly resolve the information dynamics between actin and calcium, we measured MI within each individual ROI in order to disentangle intracellular and intercellular effects. The real dataset has significantly greater MI than the randomized datasets, indicating that these two signals share information within a single cell (Figure 10C).

### 2.7. Intracellular Transfer Entropy (TE): Actin Causally Affects Calcium Dynamics, but Calcium Dynamics Do Not Causally Affect Actin Dynamics

Finally, we measured TE between the actin and calcium channels for each ROI (Figure 10C). We found that, consistent with our previous pairwise ROI data, on an individual cell basis, actin dynamics demonstrate significantly higher TE to calcium dynamics than calcium dynamics due to actin dynamics. Likewise, TE from actin to calcium dynamics is significantly higher than that of the randomized controls, while TE from calcium to actin is insignificantly different from random controls. These data further support our conclusion of a directional information flow from actin dynamics to calcium dynamics and suggest that this interaction happens at the individual cell level.

## 3. Discussion

Many aspects of information flow are consistent between actin and calcium dynamics. The modalities both display significant AIS, and both display significant MI with neighboring cells. These data suggest that both modalities are largely affected by their own histories but, at the same time, share information with their neighbors.

However, information flow patterns in actin and calcium dynamics share some key differences, indicating that they are not simply manifestations of a single signaling regime. Actin dynamics display significantly greater AIS than calcium dynamics do, while calcium dynamics display greater MI between cells than actin dynamics do. Taken together, these data point to a greater role for actin dynamics in the maintenance of a cell’s state, and calcium plays a greater role in mediating coordination between cells. This trend is further supported by our TE analysis, which shows significant information transfer in the calcium modality but not in the actin modality. Information analysis of crosstalk between the actin and calcium reveals that actin dynamics affect calcium dynamics, but calcium dynamics do not detectably affect actin dynamics (Figure 10A).

We propose (Figure 10D) that actin establishes relatively stable (high AIS) cell collectives (high MI) but does not directly mediate information flow between cells (low TE). This interpretation is consistent with the role of supracellular actin cables in tissue compartmentalization [57], and suggests that this type of compartmentalization may manifest at some level very early even in seemingly poorly organized tissue. Calcium, in contrast, does mediate information flow between cells (high MI and TE), but is less persistent than actin architecture (relatively low AIS). Finally, because actin does affects calcium dynamics (high TE, Figure 10A) we propose that actin dynamics establish compartments through which calcium mediated information flows.

Ultimately, the goal of this analysis is to inform experimental interventions to allow us to understand and control tissue behavior at the systems level. The effective information framework discussed in the introduction provides us with a quantitative way to translate our findings from simple observation to inferences of causality via future interventional experiments. Because calcium dynamics but not actin dynamics display significant intercellular transfer entropy, we predict that perturbations in one cell are likely to affect the calcium dynamics of distant cells, but not the actin dynamics. However, we predict that intervening upon actin dynamics will affect calcium dynamics, even in nearby cells, but that affecting actin dynamics should not perturb calcium signaling. Thus, the analysis that we provide here is a key step in establishing a robust, quantitative, and experimentally supported systems-level understanding of collectivity in embryonic stem cells. This case study demonstrates how the application of information theory can be used to identify new experimental interventions. Iterating the application of information theory and experimental intervention, thereby, can allow the potential for unraveling features of biological behavior across space and time that are otherwise hard to identify due to the multiscale causal interactions in complex living systems.

## 4. Materials and Methods

### 4.1. Animal Husbandry

Fertilized embryos were raised at 14 °C in 0.1X Marc’s modified ringer solution until the onset of gastrulation. Frogs were maintained in accordance with the IACUC protocol M2020-35.

### 4.2. Microinjections

Embryos were injected into 2–4 cell embryos, with *jGCaMP8S* [58] being injected at 600 ng/µL and *lifeAct-mCherry* being injected at 100 ng/µL. mCherry-Lifeact-7 was a gift from Michael Davidson (Addgene plasmid # 54491, http://n2t.net/addgene:54491, Accessed on 12 August 2022, RRID:Addgene_54491)

### 4.3. Explant Culture

Eight-chambered Nunc Lab-Tek II Chambered coverglasses were coated with 0.1 mg/mL human plasma Fibronectin (F2006, Sigma Aldrich, Burlington, MA, USA) for 60 min at 37 °C in 1X PBS [59]. Then, the dishes were washed once with PBS, once with Danilchick’s For Amy (DFA) buffer that was replaced with fresh DFA buffer [59]. The superficial ectoderm of the animal pole of Nieukoop and Faber Stage 10 embryos was surgically removed in 0.1X Marc’s modified ringer solution [60]. The deep ectoderm was then isolated and transferred to the Fibronectin coated coverglass chambers. Explants were allowed to adhere and spread overnight at 18 °C and imaged the following day.

### 4.4. Imaging

Explants were imaged on a Leica Stellaris Sp8 confocal microscope for 1 h each with a capture rate of one frame per minute. jGCaMP8S and LifeAct-mCherry were imaged in parallel with separate excitation wavelengths and separate detectors. Specific imaging conditions are available in the representative metadata file (Supplementary Methods S1).

### 4.5. Image Processing

Nine 100 by 100 pixel ROIs were selected per explant time lapse. ROIs were chosen that would remain in a single cell despite cell motion during the time lapse, and were in a domain of the cell that appeared to display actin dynamics. Each explant contained a mixture of ROIs selected at the explant periphery and more internal. An effort was made to choose ROIs from juxtaposed cells where possible with cells exhibiting prohibitive cell motion being discarded.

### 4.6. Image Randomization

Eighteen arrays of numbers 1–60 were generated in Microsoft Excel. A different array was used for each ROI, and separate arrays were used for the two channels. The 18 arrays and randomization macros are attached in Supplementary Method S2.

### 4.7. Statistics

Statistics were performed using GraphPad Prism. Pairwise significance was assayed using Mann–Whitney tests because we could not assume Gaussian distributions of our datasets. To compare real datasets to multiple randomized datasets, pairwise comparisons were used instead of ANOVA tests to prevent false positives resulting from differences between the randomized data sets, and the null hypothesis was only rejected if the real dataset was significantly different from each randomized dataset.

### 4.8. Information Theory Analysis

Active information, mutual information and transfer entropy were measured using the novel CAIM software package [55]. CAIM facilitates application of the Inform software package [56] to time series datasets.

## Data Availability

Data available on request.

## Supplementary Materials

The following supporting information can be downloaded at: https://www.mdpi.com/article/10.3390/ijms23179580/s1.
